# Suicide risk in schizophrenia: learning from the past to change the future

**DOI:** 10.1186/1744-859X-6-10

**Published:** 2007-03-16

**Authors:** Maurizio Pompili, Xavier F Amador, Paolo Girardi, Jill Harkavy-Friedman, Martin Harrow, Kalman Kaplan, Michael Krausz, David Lester, Herbert Y Meltzer, Jiri Modestin, Lori P Montross, Preben Bo Mortensen, Povl Munk-Jørgensen, Jimmi Nielsen, Merete Nordentoft, Pirjo Irmeli Saarinen, Sidney Zisook, Scott T Wilson, Roberto Tatarelli

**Affiliations:** 1 Department of Psychiatry, Sant'Andrea Hospital, "Sapienza" University of Rome, Italy; 2 McLean Hospital – Harvard Medical School, USA; 3 Department of Psychiatry, Columbia University, New York, USA; 4 New York State Psychiatric Institute, Columbia University, New York, USA; 5 Department of Psychology, University of Illinois College of Medicine, Chicago, USA; 6 Psychiatric Clinic, University Hospital Eppendorf, Hamburg, Germany; 7 Center for the Study of Suicide, Blackwood, USA; 8 Department of Psychiatry Vanderbilt University School of Medicine, USA; 9 Deptartment of Psychiatry (Burghölzli Hospital), University of Zurich, Switzerland; 10 Department of Psychiatry, Division of Geriatric Psychiatry, University of California San Diego, USA; 11 National Centre for Register-Based Research, Aarhus University, Aarhus, Denmark; 12 Unit for Psychiatric Research, Aalborg Psychiatric Hospital, Aarhus University Hospital, Aalborg, Denmark; 13 Department of Psychiatry Copenhagen University, Bispebjerg Hospital, Copenhagen, Denmark; 14 Department of Psychiatry Kuopio University Hospital, Kuopio, Finland

## Abstract

Suicide is a major cause of death among patients with schizophrenia. Research indicates that at least 5–13% of schizophrenic patients die by suicide, and it is likely that the higher end of range is the most accurate estimate. There is almost total agreement that the schizophrenic patient who is more likely to commit suicide is young, male, white and never married, with good premorbid function, post-psychotic depression and a history of substance abuse and suicide attempts. Hopelessness, social isolation, hospitalization, deteriorating health after a high level of premorbid functioning, recent loss or rejection, limited external support, and family stress or instability are risk factors for suicide in patients with schizophrenia. Suicidal schizophrenics usually fear further mental deterioration, and they experience either excessive treatment dependence or loss of faith in treatment. Awareness of illness has been reported as a major issue among suicidal schizophrenic patients, yet some researchers argue that insight into the illness does not increase suicide risk. Protective factors play also an important role in assessing suicide risk and should also be carefully evaluated. The neurobiological perspective offers a new approach for understanding self-destructive behavior among patients with schizophrenia and may improve the accuracy of screening schizophrenics for suicide. Although, there is general consensus on the risk factors, accurate knowledge as well as early recognition of patients at risk is still lacking in everyday clinical practice. Better knowledge may help clinicians and caretakers to implement preventive measures.

This review paper is the results of a joint effort between researchers in the field of suicide in schizophrenia. Each expert provided a brief essay on one specific aspect of the problem. This is the first attempt to present a consensus report as well as the development of a set of guidelines for reducing suicide risk among schizophenia patients.

## I. Background

Despite great efforts, suicide rates among schizophrenic patients remain alarmingly high. A comprehensive analysis recently appeared [1], and a number of opinion leaders have been involved in the develpment of books, papers and conferences to understand and prevent suicidal behavior in patients suffering from schizophrenia [1]. This paper is one such effort. It presents a review of the many aspects of suicidal behavior in schizophrenia and attempts to develop and share guidelines for the prevention of suicide in schizophrenics.

In 1977, Miles [2] reviewed 34 studies of suicide among schizophrenics and estimated that 10% of schizophrenic patients kill themselves. Follow-up studies have estimated that 10–13% of individuals with schizophrenia die by suicide, which is the main cause of death among these patients [3]. However, a recent meta-analysis estimated that 4.9% of schizophrenics commit suicide during their lifetime [4]. This percentage surprised many researchers as it was lower than previously thought. Regardless, it is still an unacceptably high incidence. Inskip, et al. [5] performed a meta-analysis on suicide among patients with affective disorder, alcoholism and schizophrenia and estimated that the lifetime risk of suicide was 6% for affective disorder, 7% for alcohol dependence and 4% for schizophrenia, an estimate which is consistent with Palmer's estimate. They concluded, therefore, that the lifetime suicide risk figure of 10% or more appears to be too high, although Meltzer [6] disagrees. Following an index suicide attempt, mortality from suicide in schizophrenia patients may be as high as 1% per year for the next five years [7,8]. Pompili, et al. [9] reviewed the literature on suicide among inpatients with schizophrenia and found that the suicide rate in cohorts of schizophrenic patients who were followed-up after the first hospitalization for periods ranging from 1 to 26 years was 6.8%.

Harris and Barraclough [10] included 28 studies in their meta-analysis and found that the risk of suicide among patients diagnosed with schizophrenia exceeded that in the general population more than eight fold [SMR = 8.45, CI = 7.98–8.95]. Brown [11] found that schizophrenia was associated with excess death from both natural causes (e.g., respiratory diseases) and unnatural causes (accidents, suicide, and homicide). Suicide accounted for 12% of all deaths among schizophrenia patients and about 28% of all excess deaths. According to Brown, the excess mortality was highest in first episode or early illness phase patients, indicating a high rate of suicide early in the illness. Danish studies that assessed standard mortality ratios (SMR) in successive national cohorts suggest that the SMR may be rising in first-episode schizophrenia in Denmark [12] and falling in chronic schizophrenia [13]. At the same time, other data indicate that suicide risk may be elevated across the entire course of schizophrenia. A recent examination of the suicides of all patients with schizophrenia in Finland over a 12-month period found that fully one-third of the schizophrenic suicides were over the age of 45 [14]. Despite great efforts, both on the side of drug treatment and psychosocial strategies, the number of suicides among schizophrenic patients has remained unchanged [15], although Nordentoft et al. [16] have shown that suicide among Danish patients with schizophrenia has fallen, paralleling the reduction of suicide in the general population.

Suicide attempts, which often result in death from suicide at a later time, are common among patients with schizophrenia; about 20–40% of these patients do make suicide attempts [17-19].

Many factors associated with suicide in schizophrenia have been identified, but attempts to identify high-risk patients have so far produced too many false positive results to be clinically useful [3]. Yet, identification of risk factors is a major tactic for predicting and preventing suicide. This review is based on systematic search of the international literature as well as on the experience of scholars who are dedicated researchers in the field. Opinion leaders in this field agreed to provide a summary of the state of the art for specific aspects of the problem. This paper therefore represents the first attempt to combine the efforts of researchers into suicide in schizophrenia in order to improve the understanding of the problem.

## II. Materials and methods

We conducted careful MedLine, Excerpta Medica, and PsycLit searches to identify papers and book chapters in English during the period 1966–2006. We also performed Index Medicus and Excerpta Medica searches prior to 1966. Search terms were "suicid*" (which comprises suicide, suicidal, suicidality, and other suicide-related terms), "parasuicid*," "schizophren*," "inpatient or in-patient", and "outpatient". Each term was also cross-referenced with the others using the MeSH method (Medical Subjects Headings). Using the same databases and methods, we also crossed-referenced the above-mentioned terms with key words such as "neurocognition" or "neurocognitive," "neuroleptics or antipsychotics" (all terms belonging to the neuroleptics or to the antipsychotics categories were checked).

In this way, the entire literature on suicide in schizophrenia was carefully reviewed. By reviewing selected articles we identified some specific fields of interest. Sources of information also included original epidemiological research by the authors as well as classifications and data from World Health Organization. The authors agreed on a number of key topics relevant to the aim of this paper.

## III. Results

### 1. Risk factors

There is almost total agreement that the schizophrenic patient who is more likely to commit suicide is young, male, white, and never married, with good premorbid function, post-psychotic depression and a history of substance abuse and suicide attempts. Hopelessness, social isolation, hospitalization, deteriorating health with a high level of premorbid functioning, recent loss or rejection, limited external support, and family stress or instability are important risk factors in schizophrenic individuals who commit suicide. These patients usually fear further mental deterioration, and they show either excessive treatment dependence or loss of faith in treatment. Awareness of the illness has been reported as a major risk factor among schizophrenic patients who at risk of suicide. Protective factors also play an important role for assessing suicide risk and, therefore, should be carefully evaluated. Although there is a general consensus on these factors, proper knowledge and, therefore, early recognition of patients at risk is still lacking in everyday clinical practice.

**Figure 1:**
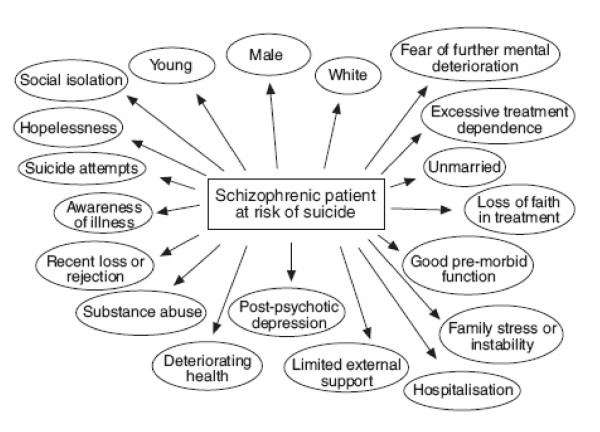
A summary of risk factors for suicide in schizophrenia.

Fenton et al. [20] and Fenton [21] described the high risk patient as a young male, with a history of good adolescent functioning and high aspirations, late age of first hospitalization, higher IQ, with a paranoid or non-deficit form of schizophrenia, who retains the capacity for abstract thinking and who may be painfully aware of the impact of a deteriorating illness on his aspirations and life trajectory. Risk factors for schizophrenia are summarized in Figure 1 and Table 1.

**Table 1:** Risk factors for suicide in schizophrenic outpatients and inpatients (modified from [9])

White, young, male (often under 30 years)
Unmarried
High premorbid expectations
Gradual onset of illness
Social isolation
Fear of further mental deterioration
Excessive treatment dependency
Loss of faith in treatment
Family stress or instability
Limited external support
Recent loss or rejection
Hopelessness
Deteriorating health
Paranoid schizophrenia
Substance abuse
Deliberate self-harm
Unemployement
Chronicity of illness with numerous exacerbation
Family history of suicide
Pre-admission and intra-admission suicidal attempts
Agitation and impulsivity
Fluctuating suicidal ideation
Extrapiramidal symptoms caused by medications
Prescription of a greater number of neuroleptic and antidepressants
Increased length of stay, increased number of ward changes, discharge planning and period following discharge
Period of approved leave
Apparent improvement
Past and present history of depression
Frequent relapses and rehospitalization
Longer hospitalization periods than other psychiatric inpatients
Negative attitudes towards medication and reduced compliance with therapy
Living alone before the past admission
Charged feelings about their illness and hospital admission
Early signs of a disturbed psychosocial adjustment
Dependence and incapability of working
Difficult relationship with staff and difficult acclimation in ward environment
Hospitalization close to crucial sites (big roads, railway stations, rivers, etc).

Positive symptoms are generally less often included among risk factors for suicide in schizophrenia. However, a number of studies have found that the active and exacerbated phase of the illness and the presence of psychotic symptoms [14,22-24], as well as paranoid delusions and thought disorder [25,26], are associated with a high risk of suicide. Patients with the paranoid subtype of schizophrenia are also more likely to commit suicide [27,20]. Suicides as a result of command hallucinations, although rare, have been reported in the literature [28]. Kelly, et al [29] reported that a large proportion of their schizophrenic patients who committed suicide had poor control of thoughts or thought insertion, loose associations and flight of ideas as compared to those who died by other means of death.

A recent systematic review of risk factors for schizophrenia and suicide [30] identified 29 relevant studies and 7 robust risk factors including previous depressive disorder (OR = 3.03, 95% CI = 2.06–4.46), previous suicide attempts (OR = 4.09, 95%CI = 2.79–6.01), drug misuse (OR = 3.21, 95%CI = 1.99–5.17), agitation or motor restlessness (OR = 2.61, 95%CI = 1.54–4.41), fear of mental disintegration (OR = 12.1, 95%CI = 1.89–81.3), poor treatment adherence (OR = 3.75, 95%CI = 2.20–6.37), and recent loss (OR = 4.03, 95%CI = 1.37–11.8). A reduced risk of suicide was associated with hallucinations (OR = 0.50, 95%CI = 0.35–0.71. The authors argued that command hallucinations were not an independent risk factor, but they increased the risk in those already predisposed to suicide. Overall, suicide was less associated with the core symptoms of psychosis and more with affective symptoms, agitation, and awareness that the illness was affecting mental function.

The neurobiological perspective offers a new approach for understadinding self-destructive behavior among patients with schizophrenia and provides a basis for screening programs other than using the risk factors that are usually part of the clinical assessment. Low concentrations of the serotonin metabolite 5-hydroxyindoleacetic acid (5-HIAA) in the cerebrospinal fluid (CSF) are associated with suicidal behavior in patients with depressive illness and with schizophrenia. In a prospective study, Cooper et al. [31] measured 5-HIAA in the CSF taken from 30 schizophrenic patients in a drug-free state and followed these patients for 11 years. Ten patients made suicide attempts during the follow-up period. The suicide attempters had significantly lower concentrations of CSF 5-HIAA at initial evaluation than the non-attempters. These findings provided evidence for an association between serotonergic function and suicide and suggested a role in schizophrenia for drugs with serotonergic effects. Hormones known to be under serotonergic control, such as prolactin (PRL), can be measured in peripheral blood after stimulation or inhibition of the serotonergic (5-HT) receptors. Fenfluramine (FEN) is a widely used serotonin probe. In humans, D-fenfluramine (D-FEN), given orally, results in an increase in plasma PRL level, which is considered to be a higly specific test of serotonergic function [32]. It has been demonstrated that a blunted PRL secretion in response to D-FEN is associated with suicidal behavior in schizophrenic patients [33]. This is an important tool since this technique gives a specific indication of serotonergic function, and it can be combined with new neuroimaging paradigms such as PET and SPECT, providing images of seronergic function in vivo [34-37].

Plocka-Lewandowska et al. [37] found an association between results of the dexamethasone suppression test (DST) and suicide attempts in schizophrenic patients, suggesting a possible association between a hyperactive hypothalamo-pituitary-adrenal (HPA) axis and suicidal behavior in schizophrenic patients. Jones et al. [39] found that nonsuppression in the DST was associated with suicidal behavior in a sample of schizophrenic patients, and non-suppression of the DST differentiated suicide attempters from non-attempters. Reports of an association between both REM sleep abnormalities and the results of the DST and suicidal behavior in schizophrenia have been reported [38,39]. Keshavan et al. [38] found that those schizophrenic patients who exhibited suicidal behavior had increased overall REM activity and REM time. Lewis et al. [40] contradicted these findings and reported that, in their sample of schizophrenic patients, total REM sleep time was associated with suicidal behavior. These authors suggested that, since serotonergic functions act to suppress REM sleep, reduced serotonergic function in schizophrenia could explain the association between suicidal behavior and REM time/activity observed by other authors. Hinse-Selch et al. [41] investigated the effects of clozapine on sleep in a sample of schizophrenic patients and found a significant clozapine-induced increased in non-REM sleep in patients who do not experience clozapine-induced fever; while the amounts of stage 4 and slow-wave sleep decreased significantly. These findings might explane the anti-suicidal role of clozapine since increasing REM sleep has been correlated with increased suicide risk.

#### a. Suicide attempts

Compared with suicide attempts among persons without schizophrenia, attempts among those with schizophrenia are serious and typically require medical attention. Suicidal intent is generally strong, and the majority of those who attempt suicide have made multiple attempts. In addition, the methods used to attempt suicide are considered more lethal than those used by suicidal persons in the general population. Gupta and colleagues [42] reported that, in their sample of patients with schizophrenia, suicide attempts were associated with the number of lifetime depressive episodes, and depression has been recognized as a major risk factor among persons with schizophrenia who have attempted suicide. Roy and associates [43] found that significantly more of their sample of patients with schizophrenia who had attempted suicide had suffered from a major depressive episode at some time during their illness.

In contrast, Drake et al. [44] found, in their sample of schizophrenic patients, that those who had attempted suicide were trying to manipulate others, consolidate support or gain entrance to the hospital. Attempts frequently occurred in the context of interpersonal conflict, such as arguments with family or housemates. These authors suggested that impulsive attempts were associated with the dysphoric side-effects of the medication, such as akathisia. Nevertheless, in a recent study, akathisia was not linked to suicidality or depression among patients with treatment-resistant schizophrenia [45].

In a study [46] comprising 500 patients affected with schizophrenia and/or affective disorders, a history of suicide attempts was associated with comorbidity, low scores on the Global Assessment Scale (GAS), low age at onset and poor premorbid adjustment. This study showed that men affected with schizophrenia were less likely to attempt suicide when compared to men with diagnoses other than schizophrenia. Among women, suicide attempts were more common in those with lower age at onset and who had no children. Kelly et al. [29] found that, among their sample of schizophrenia patients who had committed suicide, some 93% had engaged in previous suicidal behaviors versus only 23% of the patients who died by other means of death.

Suicide attempts are a significant risk factor for suicide and are associated with significant medical costs and, for this reason, an examination of risk factors for attempted suicide in schizophrenia is important. A recent systematic review of the risk factors for attempted suicide in schizophrenia identified only 14 studies that met selection criteria [47]. These authors examined 29 variables that were studied in at least two or more studies and found only five significant variables: past suicidal ideation, previous deliberate self harm, previous depressive episodes, drug abuse or dependence, and a higher mean number of psychiatric admissions

Great caution is required during the period after hospital discharge because patients with schizophrenia usually experience hopelessness and demoralization during this time. For these patients, discharge often means losing the hospital environment and the people who in some way have become central in their life. The number of psychiatric admissions, which are usually higher among patients who have attempted suicide, may be indicative of a severe relapsing illness.

#### b. Insight and suicide risk

The concept of insight has always been an important part of clinical psychiatry and neuropsychiatry nomenclature but, until recently, the term had been used to describe a disparate and wide range of phenomena [48]. During the last fifteen years, most researchers have defined insight as being comprised of at least three domains: awareness of the illness, awareness of the need for treatment, and awareness of the consequences of the disorder [49]. Increased agreement on terminology and phenomenology and the development of reliable and valid measures of insight has led to an explosion of research in this area. The relationship between insight and suicide has been an area of study that has benefited.

Many scholars and clinicians have proposed a relationship between insight and suicidal behavior in patients with psychotic disorders. Early empirical studies on the predictors of suicidal behavior in patients with psychotic disorders often noted the consequences of a fuller understanding of the implications of having a psychotic disorder, and the sense of resignation and hopelessness that was often associated with this awareness. Studies by Farberow, Shneidman and Leonard [50], Warnes [51], and a series of studies by Drake and colleagues in the 1980's [52-55] all reported very similar findings and cited a hopeless awareness of the severity of their psychopathology as one of the most important predictors of completed suicide in patients with psychotic disorders. While these studies suggested increased awareness of one's illness was associated with suicidal behavior in these patients, it was not possible to determine whether insight was directly related to suicide or only indirectly related via its influence on hopelessness. In addition, because these studies predated advances in research methodology, poor reliability for the measurement of insight contributed to the ambiguity of the results. With the development of reliable and valid measures for the assessment of insight [56-58], more recent research has been able to clarify these relationships.

Two recent studies studied the relationship between insight and suicide while taking hopelessness into account. In the first study, Kim et al. [59] compared two groups of patients with schizophrenia: 200 with a lifetime history of suicidal ideation and/or attempts and 133 without any history of suicidality. The group with a history of suicidality had significantly higher levels of both general awareness of illness and hopelessness. However, when hopelessness and insight were entered into a multiple regression model, along with several other variables, only hopelessness was statistically significant. In the second study, Bourgeois and colleagues [60] analyzed data from 980 patients from the International Suicide Prevention Trial (InterSePT) [61]. The results were similar to those of Kim et al. [59]. Greater awareness of illness significantly predicted suicide risk when entered independently into the model (with better insight associated with increased suicide risk), but was no longer significant once hopelessness was entered into the equation. Interestingly, the baseline level of awareness was associated with increased risk for suicidal behavior, but improvement in awareness over the follow-up period was associated with reduced risk for suicidal behavior. In summary, research to date suggests that awareness of illness is indeed associated with increased suicide risk in this population, but only if that awareness leads to hopelessness. This conclusion is consistent with the literature demonstrating the relationship between hopelessness and suicide [62-64] and helps to reconcile those research findings with the positive prognostic implications of improvement in awareness of the illness [65]. The severity of the hopelessness that a person with schizophrenia experiences seems contingent, at least in part, on the level of premorbid functioning and the magnitude of the decline in functioning relative to that premorbid capacity.

Several points can be made about the clinical implications of these findings. Patients with schizophrenia need to be carefully assessed for hopelessness and suicidal ideation throughout the course of their illness, particularly if there is marked improvement their in awareness of any facet of the illness syndrome. In addition, although improvements in insight are often strongly related to improvements on many clinical dimensions, we must work judiciously when we strive to increase insight in patients with other risk factors, such as young age and a substantial decline from the premorbid level of functioning. There is often a mourning process that individuals diagnosed with schizophrenia must pass through as they come to terms with what was lost with the onset of their illness, with the magnitude of the loss being determined by many factors [66]. By being attentive to this process, we can better assess the relative risk for our patients on an individual on a case-by-case basis.

#### c. Depression and hopelessness

Depression, as a mood or a syndrome, is frequently present in people with schizophrenia, and yet depression is also frequently under-diagnosed and under-treated. Depression is considered to be a major risk factor for suicidal behavior across populations. Researchers have suggested that depression can serve as a stressor or trigger for suicidal behavior among individuals who are at risk for suicidal behavior [67], and this has been demonstrated among individuals with schizophrenia [68]. For example, Harkavy-Friedman and colleagues [68,69] demonstrated that major depression serves as a trigger for suicide attempts, and depressed mood and hopelessness are correlated with current suicidal ideation.

Many researchers have found high rates of major depressive disorder among individuals with schizophrenia [54,55,69-72], and it is a requirement for the diagnosis of schizoaffective disorder in the DSM-IV [73]. In addition, many researchers have identified depressed mood and hopelessness as an important component of suicidal behavior [53,74-76]. Despite this knowledge, depression is often ignored and untreated among individuals with schizophrenia, leading to increased risk for suicidal behavior. It has been demonstrated that antidepressants can be used effectively for treating depression without increasing psychotic symptoms [77,78], but they are still under-utilized in this at-risk population.

While depression can often be masked or confused with the negative symptoms or side-effects of medication [79,80], an astute clinician can identify depression by asking targeted questions. While not all suicide attempts and completed suicides in schizophrenia are triggered by depression, psychological and psychopharmacological treatment of depression is likely to play an important role in preventing suicidal behavior in schizophrenia.

Adequate attention to depression, in the form of assessment and treatment, as well as consideration of other factors that may trigger suicidal behavior in schizophrenia, is important. Ongoing clinical assessment for the signs and symptoms of depression is essential. When identified, depression must be treated, and psychopharmacological, as well as cognitive-behavioral and psychosocial interventions, ought to be considered.

The depression-related aspects of schizophrenia are generally differentiated according to the time at which they occur during the psychotic episodes – contemporaneously with the psychosis or as a "post-psychotic depression" phenomenon. This latter syndrome has been reported as particularly relevant for suicide risk [81,82].

In general, for a variety of populations, both normal and disturbed, the most powerful predictor of suicidality, both completed suicide and attempted suicide, is depression, both the psychiatric diagnosis (major depressive disorder or biopolar disorder) and the mood as assessed by clinical judgment or by self-report inventories [83]. Beck et al. [84] found that the cognitive component of depression, which they first called pessimism and later hopelessness, was a more powerful predictor of subsequent suicide than the more general syndrome of depression. For example, in a follow-up study of psychiatric outpatients, Beck and his colleagues [85] found that hopelessness scores were significantly related to subsequent completed suicide.

Nordentoft et al. [86] studied patients with first-episode schizophrenia-spectrum disorders for one year, during which time 11% attempted suicide. Suicidal ideation and plans in the prior year were predicted by hopelessness scores, while actual suicide attempts in the prior year were predicted by both depression and hopelessness scores. Drake and Cotton [87] compared 15 schizophrenic inpatients who completed suicide subsequently with schizophrenics who did not do so during a 3 to 7 year follow-up. The suicides were judged to be more hopeless but not more depressed. Schizophrenics with depressed mood had a probability of 0.22 of subsequently completing suicide while schizophrenics with depressed mood and hopelessness had a 0.37 probability of doing so. A depressed mood alone resulted in a 0.07 probability of subsequent completed suicide and no depressed mood (with or without hopelessness) a 0.06 probability. It appears, then, that hopelessness was an important factor in predicting suicide.

Hopelessness plays a larger role in schizophrenia than its association with suicidality. For example, Aguilar et al. [88] observed that first-episode schizophrenic patients had higher levels of hopelessness (as measured by Beck's hopelessness scale) than other non-affective psychotics. Furthermore, higher hopelessness scores predicted a worse short-term outcome, in particular, worse global functioning at a one-year follow-up. (Depression scores did not predict outcome.)

Some investigators have drawn attention to the role of insight or awareness of their disorder (and its progression) as affecting the level of hopelessness and suicidality in schizophrenics. For example, Strauss [89] interviewed schizophrenics about the course of their disorder, and he noted that a relapse after gradual improvement can lead to extreme despair in patients. It appears also that insight into their disorder appears to increase the level of hopelessness in schizophrenics and increases their risk of suicide, whereas neurocognitive deficits that impede awareness reduce the risk of suicide.

#### d. Symptoms and subtype

Are there clinical symptoms or illness subtypes that are associated with suicide and that could serve as indicators of suicidal danger? Some symptoms are generally indicative of suicidal danger regardless of the diagnosis. Depressive symptoms have already been addressed, but they frequently coexist with anxiety symptoms [90,91]. Anxiety contributes to suicidality in post-psychotic depression [92], and comorbidity with panic attacks was associated with higher suicide rates in patients with schizophrenia [93]. Suicide was correlated with psychomotor agitation and restlessness [30,94] and a fear of mental disintegration, if present, predicts suicide with an odds ratio of 12.1 [30]. Akathisia is manifested subjectively in an unbearable feeling of inner tension and restlessness, and subjective awareness of akathisia is also associated with higher suicidality. Findings from a study devoted to this topic demonstrated that, among patients with akathisia, there was a greater likehood of suicidal behavior than among those without akathisia [95]. These authors stressed that their findings imply that the suicidality may be related to internal feelings of distress that are concomitantly expressed both as subjective restlessness and as hopelessness and suicidal ideation. Akathisia is also associated with a constellation of symptoms with both affective and anxious features as well as motor components.

In addition to general risk factors, there may also be risk factors more or less characteristic for patients of a particular diagnostic group. Are there specific characteristics of the schizophrenic disorder associated with or predisposing to suicide? Separate sections of this review are devoted to the role of positive symptoms, negative symptoms, command hallucinations and insight. According to Zilboorg [96], clinical evidence for strong hostility can be found in every suicide, and aggressiveness, impulsivity and non-compliance are particularly frequent in schizophrenic illness. These characteristics help to differentiate between suicidal and non-suicidal schizophrenia patients [97]. Hostility at admission was associated with long-term suicide risk [21], and involvement of the police at the time of admission seems to be a specific risk factor within the schizophrenia population not encountered elsewhere [98]. However, it is perhaps impulsivity rather than aggressiveness that may be of importance. Suicidal subjects were found to exhibit acting-out behavior, to run away from hospital and to be more often discharged against medical advice [24]. Many suicide victims experienced compulsory hospital treatment, and the majority of them had poor treatment adherence [24,99].

The importance of psychopathology for suicidal behavior may change over time. Considering the condition of the patient immediately before suicide, no uniform picture could be identified. A withdrawal from relationships due to depression has been described, as has an increase in the patient's paranoid behavior, and both should be regarded as acute signals of suicidal danger [25]. Farberow et al. [100] described presuicidal schizophrenic patients as extremely tense, restless and impulsive. Such patients can suddenly become quiet and calm at the time the decision to commit suicide is made. A comprehensive account of the psychopathological conditions preceding suicide has been provided by Wolfersdorf et al. [101]. In comparison to schizophrenic controls, suicides had a higher degree of subjective suffering and ambivalence, and most of them were preoccupied by the feeling of having failed. According to Drake et al. [102], the patients' presuicidal condition is characterized by feelings of inadequacy, hopelessness and fears of mental disintegration. Also, the patients tend to develop a more negative or indifferent attitude towards the psychiatric personnel, and they often no longer request support or attention [103].

Schizophrenia is an illness of considerable heterogeneity, and several attempts have been made to differentiate subtypes. Regarding suicide, classical subtypes of paranoid, catatonic, hebephrenic, and undifferentiated schizophrenia do not seem to be of importance [94,104]. Andreasen and Olsen [105] proposed differentiation into positive, negative and mixed schizophrenia. There is some evidence for a weak negative correlation between positive symptoms, and thus positive schizophrenia, and suicide [30]. Another typology has been devised by Crow [106] who differentiated the type I schizophrenia syndrome, equivalent to acute schizophrenia, and type II, equivalent to the defect state. Both an early onset of a defect state [24] and the deficit subtype of the illness [20,21] were associated with a reduced risk of future suicide. Nevertheless, it is not the specific syndrome, but the course of the illness, frequent relapses [24,101], a high severity of illness, a downward shift in social and vocational functioning [21,107,108], and a realistic awareness of the deteriorative effect of the illness that are the schizophrenia-specific suicide risk factors [3].

There are many ways to classify suicidal patients, and many of these typologies are also applicable to patients with schizophrenia. For instance, a differential typology has been proposed with respect to the "hard" and "soft" suicidal method [109], an ethical typology based on the role a clinician may play in the suicidal process [110], and a sociological typology reflecting the societal level of social integration and moral regulation [111]. The clinical usefulness of all these typologies for predicting suicide seems to be limited, however, and the same applies to the differentiation between single suicides, extended suicides and suicidal pacts. Both latter types are extremely rare in patients with schizophrenia.

About one third of suicide victims are found to meet the criteria for a personality disorder [112], and a classification using the presence or absence of Axis II disorders would be feasible. Nevertheless, this variable seems to play a less important role in schizophrenia due to its less frequent comorbidity with schizophrenia. In contrast, comorbidity of schizophrenic and substance use disorders is very frequent [113], and a typology based on the additional presence or absence of an addictive disorder could be meaningful, the more so as drug misuse or dependence considerably increases the risk of suicide [30].

Some other suicide subtypes have been described in schizophrenic disorders, but they have been only clinically inferred and not empirically tested. Based on their study of psychotic inpatients and their behavior in the psychiatric hospital setting, Farberow et al. [100] proposed three subtypes of schizophrenic suicide: (1) the unaccepting, grossly disturbed patient resisting hospitalization; (2) the dependent, satisfied patient whose suicide outside the hospital appears to be a consequence of stressful conflict and ambivalence concerning the home environment; and (3) the dependent, dissatisfied, demanding patient who has no other place to go and yet seems to have lost faith in the therapeutic potential of hospitalization. In an investigation on suicide [114], the authors learned to differentiate two other clinical types of schizophrenic suicide: (1) Type I schizophrenia suicide, characterized by early illness onset along with early difficulties in psychosocial adaptation, and (2) Type II characterized by a later illness onset where the patients often show a high premorbid functional capacity. However, due to the seriousness of their illness, they experience a distinct psychosocial and professional downward mobility. Patients of both types have insight with regard to their condition and are capable of critical and realistic self-assessment of their reduced life perspectives [115]. Their suicide occurs in a non-psychotic condition. Type I patients realize their failure in comparison with the achievements of their peers, while Type II patients are not able to live up to their high expectations and feel inadequate in relation to their own goals [102]. In both types, suicide appears to be the result of a realistic appraisal of the patients' whole life situation including the incapacitating illness and its negative psychosocial consequences.

##### Positive and Negative Symptoms as Suicide Risk Factors in Schizophrenia and other Psychiatric Disorders

The relationship between suicide and psychiatric disorders has remained an important question over the past three decades in psychiatry and psychology. A number of classic studies have attempted to connect suicide to a general history of mental illness and to the specific diagnoses of depression, alcoholism, schizophrenia, and organic psychoses [116-119]. However, as Hendin [120] pointed out, "the vast majority of depressed, schizophrenic, alcoholic or organically psychotic patients do not commit or even attempt suicide." Hendin went on to suggest that "the interest in classifying populations of suicidal patients by their psychiatric diagnoses is being supplemented by an interest in understanding what makes a minority of patients within any given diagnostic category suicidal while the majority are not suicidal."

The search for suicide risk factors independent of diagnosis has been espoused by a number of researchers and clinicians representing several different points of views. Weismann et al. [121], for example, suggested that suicidal patients exhibited greater hostility than did depressed patients. Beck and his colleagues [76,122] found that hopelessness was a stronger predictor of suicide than the degree of depression. Fawcett et al. [71] argued that different risk profiles may emerge for different diagnoses.

The differentiation of positive and negative symptoms has become a key factor in understanding psychiatric disorders and the potential differences between various types of psychiatric disorders. Positive symptoms refer to flagrant reality distortions such as psychosis (e.g., delusions and/or hallucinations) and disorganization/formal thought disorder. Negative symptoms refer to symptoms such as poverty of speech and flat affect. A third type of symptom grouping involves neurocognitive disorders or cognitive deficits (e.g., concrete thinking and slow processing speed).

The distinction between positive and negative symptoms was made originally by Hughlings Jackson [123]. Kraepelin's [124] seminal formulation viewed the disorder that we now label as "schizophrenia" as an early-onset dementia marked by a deteriorating clinical course. Although Kraepelin [124] emphasized both positive and negative symptoms, the attention of both researchers and clinicians was drawn to the most flagrant and dramatic positive symptoms – hallucinations, delusions and disorganization/formal thought disorder – as the principal components of schizophrenia [125]. In the last three decades, there has been renewed interest by investigators in the distinction between positive and negative symptoms [126-131], and specifically in the examination of the more stable negative symptoms associated with schizophrenia such as poverty of speech and flat affect. There has also been increased interest in neurocognitive impairment or cognitive deficit symptoms such as slow processing speed and concrete thinking [132].

There have been a few studies exploring the relationship between positive symptoms and suicidal activity. For example, there is strong evidence that psychotic episodes precipitate suicide attempts (and homicide) in some schizophrenic persons [133,134]. Several interesting studies have explored the relationship between type of delusional content and serious suicide attempts [135,136]. There have been fewer studies on the relationship between negative symptoms and suicide. For example, Fawcett et al. [137] found a relationship between anhedonia and committing suicide within one year.

Two recent studies by Kaplan and Harrow [138,139] and a review article by Kaplan et al. [140] have explored the relationship of positive symptoms, negative symptoms, cognitive deficits and overall post-hospital functioning to subsequent suicidal behavior at a two-year follow-up of psychiatric patients. The sample of 203 patients from the Chicago Follow-up Study included 71 patients with schizophrenia, 35 with a schizoaffective disorder and 97 with non-psychotic depression. The results supported a multifactor model of suicide risk. Some risk factors held across diagnosis (e.g., poor early functioning) while others were diagnostic-specific: Early psychosis predicted later suicidal activity for both schizophrenia and schizoaffective patients but not for depressives, and some negative symptoms predicted later suicidal activity for schizoaffective patients while some cognitive deficits predicted later suicidal activity for non-psychotic depressives. The effects of psychosis were almost totally mediated through the level of functioning for the schizophrenia patients but not for the schizoaffective patients, for whom psychosis directly affected later suicidality independently of the effects of poor functioning.

The results of this study begin to establish a tentative basis for a disease-based approach to suicide prevention. A suicide prevention approach for schizophrenia patients should center on improving their over all functioning and decreasing their general discouragement and hopelessness. Treatment for the schizoaffective patients in contrast should focus additionally on the reduction of psychosis *per se *in addition to the reduction of negative symptoms. For non-psychotic depressive patients, the reduction of cognitive deficits may be especially important in preventing later suicidal activity independent of the improvement in overall functioning. Clinicians should consider assessing hopelessness and demoralization in all diagnostic groups to help evaluate potential suicidal risk activity.

##### Command hallucinations

Command hallucinations, wherein patients hear voices explicitly instructing them to engage in specific acts [141], are more common among those with schizophrenia-spectrum disorders than is often recognized, occurring in 18–50% of that population [28,142]. Often these command hallucinations are suicidal in nature, thereby placing individuals who are vulnerable to suicide at even greater risk.

However, there are few empirical studies in this area, and their results are conflicting as to the legitimacy of command hallucinations as a consistent risk factor in suicide or violence toward others. Hellerstein et al. [141] conducted one of the first controlled studies investigating the relevance of command hallucinations in suicidal behavior or violence. Comparing patients with and without command hallucinations yielded no significant differences in rates of suicidal or assaultive acts. More broadly, patients with hallucinations (regardless of type) were just as likely to report suicidal ideation as those not experiencing hallucinations. Zisook et al. [28] similarly reported that patients with command hallucinations and those without command hallucinations did not differ on number of prior suicide attempts, nor on a history of violent/impulsive acts. A literature review by Rudnick [143] also showed a lack of a relationship between command hallucinations and violence toward self or others. More recently, Harkavy-Friedman et al. [120] sampled 100 inpatients with schizophrenia or schizoaffective disorder, divided between those who had experienced command auditory hallucinations (n = 22) and those who had not (n = 78). The rate of suicide attempts did not differ significantly between the two groups.

On the other hand, Rogers et al. [144] compared 56 forensic patients with a lifetime history of command hallucinations with 54 non-command hallucinators. The presence of self-injurious command hallucinations was a significant predictor of self-harming behavior, although this study was not restricted to schizophrenic patients. Furthermore, Nordentoft et al. [84] reported that hallucinations were one of only two significant variables predicting attempted suicide in a randomized controlled trial of integrated treatment for patients with schizophrenia-spectrum disorders.

The aforementioned study results indicate that the prognostic significance of command hallucinations is unresolved. Some researchers cite a connection between command hallucinations and various forms of violence, whereas others find no empirical evidence of a relationship. Even in the midst of this uncertainty, there are several points upon which many studies agree: (a) that the rates of occurrence for command hallucinations is high [145], (b) that such symptoms are vastly underreported [146], and (c) that command hallucinations hold clinical significance for violence even in the absence of statistical significance [28,142,144].

These conflicting research findings are probably the result of the methodological problems inherent in this type of research: underreporting of the symptoms [28,146]., small sample sizes [3,121], and a lack of standardization in defining suicidal behavior or the presence of hallucinations. Specifically, the type of hallucination has not always been clearly stated in the studies, leaving readers unclear about whether patients were experiencing violent, suicidal, or benign command hallucinations. Research also faces the problem of knowing whether patients were actively hallucinating during the behavior being studied (suicidal or violent behavior) [147]. Furthermore, researchers in the past have sampled diagnostically heterogeneous groups, mixing schizophrenia with bipolar disorders, personality disorders, and severe mood disorders [143-145] These results have then been compared, perhaps unfairly, to studies that sampled only people with schizophrenia [143,148,149]

Thus, command hallucinations occur more frequently than is often recognized and hold potentially vital clinical significance. In order to prevent suicide, direct screening for command hallucinations should be incorporated into any suicide assessment within this patient population.

#### e. Comorbid substance use disorders

Substance use/abuse/dependence is often comorbid with schizophrenia, and psychosis and substance use are both found to increase suicide risk [150]. Researchers, in studies of two American cohorts, found significantly more comorbid substance abuse among people with schizophrenia who were suicidal, particularly among the younger ones [151-153]. They stated that it is important, in view of the changing patterns in the epidemiology of schizophrenia comorbid with substance use/abuse, that clinicians obtain accurate drug-use history in order to detect and promptly treat drug use/abuse. Youths who abuse drugs are at increased risk for committing suicide, and drug or alcohol abuse is found in about 70% of children and adolescents who commit suicide [154].

Harris and Barraclough's [10] meta-analysis on suicide as outcome in mental disorders reported on the standardized mortality ratio (SMR) for various psychoactive substance-use disorders. After combining the studies, they compared suicide risks of drug users and nonusers and found the SMRs for suicide of users to be higher than those of nonusers in all groups. In subjects with alcohol dependence and abuse it was 6-times higher, in opioid dependence and abuse 14-times, and in cannabis users 4-times. In this meta-analysis, suicide risk among schizophrenic patients was 8.5 times greater than among nonschizophrenics. Subsequently, Wilcox et al. [155] located twenty studies not included in the Harris and Barraclough [10] review and identified another 22 studies published after 1997. By combining data from all of these studies, they found more robust associations between suicide and overall opioid use disorder, mixed intravenous drug use, alcohol use disorders among women.

The increased suicide risk in substance-abusing schizophrenic patients [156-162] could be the result of a cumulative effect of many factors or events, such as the loss of remaining social control through the consumption of psychotropic substances, noncompliance with antipsychotic medication, and presence of paranoia and depression [163]. In Allebeck and Allgulander's [164] sample of young male substance abusers, the diagnostic category associated with the highest suicide risk was schizophrenic psychosis. Abuse substances worsen both symptoms and prognosis of the illness and are related to higher relapse rates.

Suicide may become the ultimate solution for reducing suffering caused by hopelessness and social isolation. Various studies have recognized the importance of substance abuse in the suicides of patients with schizophrenia [165-169]. Drug and alcohol abuse increase the risk of suicide in the general population [151,170-173] and, when this behavior is associated with a diagnosis of schizophrenia, the risk is much higher. It is also important to take into consideration the difficulties in reaching marginalized individuals. A comparison of patients who began drug abuse before their first admission with those who began abusing drugs after their first admission showed that the use of specific drugs was associated with significant differences in age, age at first hospitalization, premorbid functioning and subtype of schizophrenia. The differences were not uniform across the different drugs [174].

But, when comparing schizophrenics who attempt suicide with nonattempters, drug abuse is not found to differ between the two groups [69]. However, schizophrenic patients who use substances do have more positive symptoms, especially hallucinations [175], and more suicide attempts than patients with the same diagnosis and no substance use [175,176]. Interestingly, hallucinations [142], but not delusions [177], were found to increase the incidence of suicide attempts in patients with schizophrenia, independently from alcohol/drug abuse/dependence [142].

#### f. Suicide risk during adolescence

The suicide risk for adolescents or young adults with schizophrenia is three times higher than that for adult schizophrenic patients. The first two years of the disease are especially dangerous. Suicidality in this group of young patients often goes along with the harmful use of psychotropic substances and affective syndromes [178]. Among patients with psychotic symptoms, the risk of suicidal behavior is significant higher in cohorts that include adolescents and young adults as well as older paitents.

The situation of individuals with first-episode schizophrenia in life is often much more unstable since they are not used to the disorder and since, as adolescents, they are facing the typical problems and conflicts of young persons beginning a new phase in life. They are confronted with a painful psychological crisis with two aspects, and the symptoms of psychosis might be only a part of this crisis. In addition other syndromes, such as mood disorders and addictive behaviors, complicate the situation and increase the risks for the individual.

Though various approaches for first-episode schizophrenia have been developed in recent years, it is still difficult for a person suffering from symptoms of psychosis for the first time to find appropriate support. It usually takes several months until this person is diagnosed correctly and treated by a psychiatrist. The current health-care system still fails to meet the needs of this group of patients. Early detection and intervention programs are crucial, and suicide prevention must be an important component of these programs.

#### g. Suicide risk during hospitalization

A recent Danish register-based study by Qin and Nordentoft [179] found that 37% of men and 57% of women who committed suicide had a history of admission to psychiatric hospitals. This suggests that men at risk for suicide are less likely to seek or receive psychiatric treatment, but the study confirms previous reports that suicide risk is highly associated with a history of admission to psychiatric hospital. It further showed that the risk peaked, not only shortly after discharge as reported in the literature [180-184], but also shortly after admission. For patients with schizophrenia and related disorders, there was, as in other conditions, two sharp peaks in suicide risk, the first immediately after admission (adjusted risk ratio around 80 compared with persons with no history of admission) and the second peak shortly after discharge (adjusted risk ratio around 110 compared with persons with no history of admission). Approximately one third of the suicides in schizophrenics occur during admission or during the first week after discharge. From a preventive perspective, this is actually good because it identifies important risk periods upon which preventive interventions should focus. For instance, suicide among patients with schizophrenia currently admitted or discharged within last week accounts for almost three percent of all suicides in Denmark.

It is possible that a very small proportion of the suicides registered as suicides after discharge were actually suicides committed while hospitalized if the person did not die immediately but was transferred to medical department where he or she died of the consequences of a suicide attempt carried out during psychiatric hospitalization. This concerns very few cases and does not influence the result of the analyses.

##### What are the time trends in suicide risk associated with schizophrenia?

Several papers have reported increasing risk of suicide in schizophrenia over time [185-187]. This development has been attributed to deinstitutionalization. To examine this development carefully in the Danish population, Mortensen and his colleagues [188,189] combined four longitudinal population-based registers and followed the changes in the suicide rates for patients with schizophrenia and related disorders. In 1980, the suicide rate of the general population in Denmark peaked and reached a level that was among the highest in the world, with 34 suicides per 100,000 inhabitants. After 1980, the number of suicides decreased each year, and in 1997, the rate was 15 per 100,000 inhabitants, a 56-percent reduction in the suicide rate during the period 1980–1997. In Denmark, approximately half of the persons who die from suicide have previously been admitted to psychiatric departments and more than one-fourth have been admitted during the last year [188,189]. The study investigated whether there was a decline in suicide rate among patients with schizophrenia and related disorders parallel to the decline in the Danish suicide rate from 1981 to 1997. Although the risk of suicide among patients with schizophrenia and related disorders is roughly 20 times higher than among never-admitted persons in the general population, the suicide rate among patients with schizophrenia and related disorders in Denmark declined by a half from 1981 to 1997. The change in the suicide rate among these patients was the same as the change among never-admitted persons in the general population, except that patients with non-schizophrenic psychoses in the schizophrenia spectrum had a faster decrease in suicide rate compared to the never-admitted population [190]. Thus, these data did not support the notion that deinstitutionalization in Denmark resulted in an increased suicide rate. It is unknown whether this finding can be replicated in other countries.

#### h. Medical staff and suicide risk

The situation of people immediately prior to their suicidal act is critical for its prevention. Schizophrenic patients who decide to commit suicide often contact health-care workers in the days or weeks before their act. However, many factors impair the ability of treatment professionals to recognize the acute risk of suicide in their patients. These factors are related to the suicide phenomenon itself, to problems associated with the treatment system and to the treatment practices adopted by professionals, but they are also related to the personal psychological issues of the workers [25,191,192].

Staff knowledge of suicidology and their psychological readiness to deal with the anxiety and despair of suicidal patients are important in the treatment process, and uncertainties may be fatal [193]. Increased attention to interpersonal behaviour may provide a basis for more accurate recognition and more successful long-term treatment of high-risk suicidal patients. Withdrawal by a depressed schizophrenic patient and an increase in paranoid behavior should be regarded as signals of an acutely increased risk of suicide [25]. In addition, awareness that psychological and somatic symptoms are connected could facilitate the identification of an acute risk of suicide [194].

Particular attention should be paid to the suicide risk in situations in which the treatment regimen is changed in some significant way [25,52,53,191,195-202]. Difficulty in recognizing depression in schizophrenics is further complicated by the fact that depressive withdrawal from personal relationships may be misinterpreted as a negative symptom related to the primary illness [203,204]. Organizational factors and staff turnover are also obstacles to maintaining suicide-prevention activities and making them routine in psychiatric care [205].

##### Interactional factors

Suicide often comes as a surprise to both relatives of the suicide victim and those who have treated the individual, even in cases in which the victim was known to be strongly self-destructive. The feeling of concern evoked by self-destructive persons in those with whom they are in contact disappears or is absent immediately prior to suicide. According to Tähkä [206], this is because, after the final decision to commit suicide, the person ceases to send emotional messages. When the person no longer hates anybody but himself, then someone's love and concern no longer prevents him. The narcissistic regression has reached a point at which the person has lost his object-orientedness. Loss of concern by professionals is also associated with an acute risk of suicide in depressed schizophrenics [25].

Maltsberger [207] has noted that severely self-destructive persons cannot be reached by means of empathy immediately before they commit suicide. Calming before suicide is achieved because formulating suicide plan in itself is sometimes sufficient to master the sense of intolerable helplessness [208]. Ringel [209] has described a self-destructive state using the phrase "ominous quie." In this situation, the dynamic force expresses the hidden channelling of the drives into a single direction – negation of life and self-destruction. Before complete isolation and the constriction of human relations, there is a period of dependency on one person only [209] – the chosen rescuer [210]. According to Menninger [211], there are three components in the suicidal act: the wish to kill, the wish to be killed, and the wish to die. Jensen and Petty [210] suggest a fourth element – an unfulfilled wish to be rescued. In psychotic states, the choice of rescuer can be confused, and then the opportunity for rescue may be brief. It can also be so symbolic that the fantasy of the suicidal person is imperceptible.

Ignoring the suicide risk is very common in health care professionals. Knowledge about self-destructiveness in a patient can even be repressed or denied by an experienced therapist [190]. Fear of stigmatization because of the schizophrenia and even more so because of the suicidal ideation is probably one reason that these clinical antecedents are hidden by the patient and ignored by the therapist. It is important that suicide is one of the topics discussed regularly during the treatment.

Some depressed schizophrenics, before committing suicide, complain about the treatment personnel and about their treatment in general. Meissner [213] has described the relationship between paranoid states and depression, emphasizing that those who have paranoid ideas often also have self-destructive ideas. One study has shown that paranoid ideas are a specific risk factor for suicide in psychotic patients [214]. The same association has also been found in the case of schizophrenia [26]. In "Practice guideline for the treatment of patients with schizophrenia" [215], it has been pointed out that some risk factors for suicide in schizophrenia are the same as those for the general population, and some are specific for schizophrenia. These specific factors include severe depressive and psychotic symptoms, with an increase in the patient's paranoid behavior. Accusations against personnel can be most intense immediately prior to suicide. However, at the critical moment, just before committing suicide, the patients cease complaining about staff. The role of paranoid delusions and projection as factors in increasing the risk for suicide is not always understood, but understanding their role provides opportunities for preventing suicide [25]. However, the aggression and projective defence strategies against self-destructiveness in patients are hard for even experienced professionals to tolerate.

An increase in somatic complaints may also be a sign of acute suicide risk in schizophrenia as well as in depression [194,216]. This complaining seems to represent the last attempt to establish an emotionally meaningful relationship with a care provider immediately before suicide. If a worker has identified the possibility of depression underlying the somatic symptoms but has not talked about it to the patient, he or she may not have an experience of psychologically important caring during the treatment relationship.

##### Postvention

Postsuicide prevention (postvention) should become an established treatment practice in the cases of patient suicide during health care. Postvention after the patient's suicide is an important part of the treatment relationship and of the prevention of suicide in other patients. Suicide risk assessment is the most difficult kind of assessment in psychiatric practice [193,217,218]. Furthermore, treatment professionals often seem to have great difficulties in recognizing and dealing with their own affective reactions and internal incentives [25,191,192]. Specific training and consultation in suicidology is needed, and it should address facts and provide skills for dealing with difficult emotions aroused in the encounter with suicidal patients

A feeling of guilt after a patient's suicide is common among treatment professionals. However, many survivors respond well to the concept that their feelings of guilt represent positive caring for others more than any real culpability [219]. A patient's suicide is among the most difficult professional experiences encountered by a psychiatrist [220]. Adequate supervision, debriefing and postvention should be provided [25,191,221].

### 2. Prevention and treatment of suicide in schizophrenia

#### a. Pharmacotherapy of Suicide in Schizophrenia: The Clozapine Indication

There is little evidence that the typical neuroleptic drugs, with or without antidepressants, as well as the atypical antipsychotic drugs other than clozapine, have an effect on fatal or non-fatal suicidal behavior in patients with schizophrenia [222,223]. However, there is considerable data that indicates that clozapine does reduce the risk of suicide. Clozapine was first reported to reduce the rate of suicidality in 88 patients with schizophrenia in a mirror-image study [224]. The percentage of patients with no suicidality increased from 53% at baseline to 88% during treatment with clozapine. There was an 86% decrease in suicide attempts. Nearly identical results were obtained in another mirror-image study in hospitalized patients [225].

An epidemiologic study of mortality and morbidity in current and former clozapine users based upon the US Clozaril^® ^National Registry reported that mortality from suicide was markedly decreased in current clozapine users in comparison with past users [226]. American and English clozapine registry data revealed a reduced risk of suicide for patients treated with clozapine compared to the general population of patients with schizophrenia [227,228].

However, there are limitations in these studies that limit the confidence that the findings reach the highest standards of evidence-based medicine, such as no randomization of the patients in the treatment groups and the use of retrospective, broad inclusion criteria. These issues were addressed in the International Suicide Prevention Trial (InterSePT), a randomized, two year, open-label trial with blind ratings, and determination of whether potential endpoints met criteria for a suicide attempt or a hospitalization to prevent suicide by a blind, independent, expert Suicide Monitoring Board (SMB; Meltzer et al. [61,229]. It included 980 patients with schizophrenia or schizoaffective disorder who were at high risk for a subsequent suicide attempt, based primarily on having made at least one suicide attempt in the three years prior to study entry or on being currently suicidal. The primary outcome measure was either time to a suicide attempt (including death by suicide) or hospitalization to prevent suicide. A significant 24% difference in the hazard ratio for this endpoint in favor of clozapine was found. The number of patients needed to be treated with clozapine in order to reduce the risk of one suicide event was 13. Clozapine was superior to olanzapine in patients with schizophrenia or schizoaffective disorder, in neuroleptic-resistant as well as neuroleptic responsive patients, and in both males and females. The two drugs did not differ in overall efficacy in reducing total psychopathology, positive and negative symptoms, or depression. Thus, the difference between the impact of the drugs on suicidality was not secondary to other efficacy differences, confirming the view of suicide as a separate dimension of the schizophrenia syndrome. As a result of this study, the Food and Drug Administration of the United States approved an indication for clozapine to reduce the risk of suicide in schizophrenia. Hennen and Baldessarini [230] recently completed a meta-analysis of available data on the issue and concluded there was a substantially lower overall risk of suicidal behavors and completed suicides for clozapine. Thus, there is strong evidence to suggest that, for patients with schizophrenia or schizoaffective disorder who have made and survived a serious suicide attempt, or who can be judged to be at very high risk for such an attempt based on careful assessment, clozapine treatment should be instituted and maintained.

#### b. Non-Pharmacological Treatment of Suicide in Schizophrenia

Draket al. [201] noted that there is a need for empathic support in reducing suicide risk. These authors suggested that clinicians should acknowledge the patient's despair, discuss losses and daily difficulties, and help to establish new and accessible goals. Social isolation and work impairment have been reported as risk factors for suicide in individuals with schizophrenia [27,53,231]. Individuals with good premorbid functioning are those more at risk of suicide. Interventions such as social skills training, vocational rehabilitation and supportive employment are therefore very important in the prevention of suicide in schizophrenic patients. Broadly speaking, these kinds of therapies focus on working out daily problems rather than achieving psychological insight. It has become increasingly clear that supportive, reality-orientated therapies are generally of great value in the treatment of patients with schizophrenia. In particular, supportive psychotherapy aims at offering the patient the opportunity to meet with the therapist and discuss the difficulties encountered in daily activities. Patients are encouraged to discuss concerns about medications and side-effects as well as issues such as social isolation, money and stigma. The therapist plays an active role as he gives suggestions and shares good and bad periods empathically. The nature of these treatments and their availability vary greatly from place to place. Psychosocial approaches have however limited value for acutely psychotic patients.

Mueser and Berenbaum [232] reviewed controlled trials of psychotherapy and concluded that reality-orientated psychotherapy is superior to a dynamic, insight-orientated approach. Nevertheless, exploratory psychotherapy may have some benefits as it gives patients who have achieved stable remission the opportunity to understand inner conflicts and discuss, within a solid therapeutic alliance, suicidal thoughts or suicidal behavior. Patients learn to dealuse symbolism and thought rather than action (suicide) [233,234]. However, any psychotherapy technique with schizophrenic patients requires certain alteration and modifications of the standard approach [235-237]. An approach elaborated by Hogarty et al. [238,239] is Personal Therapy, which includes three levels of treatment with defined criteria for progression from basic to more challenging levels. Treatment begins from early months after discharge, which aims at clinical stabilization and therapeutic joining, and moves in later phases to promoting introspection and an understanding of the relationship between stressors and maladaptive responses. An intermediate phase promotes skills remediation, relaxation training, role-playing and psychoeducation. There is evidence to suggest that the combination of psychosocial and pharmacological treatments increases compliance and helps to achieve a better outcome [240].

Cotton et al. [53] stressed the importance of psychotherapy with schizophrenic patients who are at risk of suicide and noted the need to appreciate their hopeless awareness of the chronic illness. According to Westermeyer et al [64], the surviving schizophrenic individual may be the type of patient who is able to adjust to life as a chronic schizophrenic or as a moderately and episodically impaired schizophrenic, and thus may be less likely to commit suicide.

Increased insight may parallel increased suicidality, but this is not *per se *a reason to try to decrease insight in patients with schizophrenia. In fact, insight is also positively related to compliance with treatment, both medication and psychotherapy, which both can help to reduce suicidality. Gradual increases in insight secondary to treatment were also related to decreased suicidality in one study. Dramatic increases in insight should, however, be avoided and should be managed within an appropriate therapeutic relationship. Structured psychotherapies might add to the benefits of successful drug treatment of schizophrenic patients. Thus, insight may have a bidirectional impact on suicidality. It might increase it through increased hopelessness and despair [241], and these feelings may arise because the patient realizes that he or she with have to depend on lifelong medication and/or understands the social consequences of having schizophrenia. On the other hand, gradual gains in insight brought about by successful drug treatment and/or psychotherapy may decrease suicidality and may further contribute to compliance, which is a factor that protects the patients from relapses and recurrences. In turn, the benefit from adhering to treatment may make the patient's outlook on his or her illness more positive, thereby reducing suicidality. The best way to achieve these goals may be to combine drug treatment with psychotherapy, a method that has proved to be superior to each type of treatment alone in other types of mental disorders. Controlled data in this respect, however, are lacking [82].

#### c. Changes in suicide rates

The suicide rate expresses a balance between protective and risk factors. During the last century, several measures might have influenced the suicide rate. The introduction of chlorpromazine in the 1950s made it possible to treat the psychotic symptoms of schizophrenia but, in the years after the introduction of chlorpromazine, the suicide rate actually increased. This might have resulted from increased patient insight into the illness. The patients were not racked with hallucinations or delusions, but they were still not capable of working or living without help from the community [242].

Deinstitutionalization began in the 1960s, and the number of hospital beds decreased during the following 40 years. However, the association between these changes and the suicide rate is not clear. The intent of deinstitutionalization was to improve the quality of life for patients, but it is a very difficult and demanding challenge for the society to treat patients with schizophrenia in their homes. It is not accomplished simply by closing beds. The influence of deinstitutionalization on the suicide rate is difficult to interpret because there were conflicting results [186,243,244]. The number of beds also produced conflicting results because many patients were actually not discharged to their homes but to other institutions. Thus, a trans-institutionalization occurred in many cases.

In the 1990s, the atypical antipsychotics were introduced and it seems that these drugs might have some anti-suicidal properties, especially clozapine [230,245]. This might be to due their lesser propensity to cause extra pyramidal side-effects (EPS). (There is some evidence for a relationship between suicidality and EPS [95].

Another factor influencing the suicide rate could be the introduction of the selective serotonin reuptake inhibitors (SSRI) in the 1980s because they are less toxic in overdose and because it now became easier to treat depression in patients with schizophrenia. Depression in schizophrenia is very common and is associated with suicidality [8,246].

According to the WHO, the general worldwide suicide rate has increased the last 50 years. The figures for suicide in schizophrenia are not present for many countries, but in Denmark and Norway the suicide rate in schizophrenia has been decreasing since 1990 (Gurli Perto, Danish Central Psychiatric Research Register, personal communication 2005 and Statistics Norway), paralleling canges in the general suicide rate.

## IV. Conclusions: Preventive Measures and Goals for the Future

The clinical implications of this review are that prevention is likely to result from active treatment of affective symptoms and syndromes, improving adherence to medications, and maintaining special vigilance in patients with risk factors [30]. Clinical practice guidelines have identified a number of evidence-based treatments related to reducing suicidality in schizophrenia [220].

Difficulties in assessing suicidal risk in schizophrenia are related to the phenomenon of suicide *per se*, to problems associated with the treatment system or treatment practices, and to the personal psychological issues of the workers. Suicidal acts among people with schizophrenia were reported as being often so impulsive and difficult to predict that the traditional risk scales and interviews were of limited value in a clinical assessment [247]. However, schizophrenics do communicate their potential for suicide [248]. The American Psychiatric Association's clinical practice guidelines for assessment and treatment of patients with suicidal behaviors have provided an outline and clinical details for assessing individual patients [220].

An important issue for further investigate and understand suicide in schizophrenia is family history of suicide. Such topic was investigated in several studies and results were conflicting. In a metaanalysis, Hawton[30]found that family history of suicide among patients with schizophrenia was associated with OR = 1.82, (95 % CI = 0.56–5.94), thus a non-significant finding. Roy [249] inestigated 243 patients with a family history of suicide who were compared with 5,602 patients with no family history of suicide. A family history of suicide was found to significantly increase the risk for an attempt at suicide in patients with a wide variety of diagnoses: schizophrenia, unipolar and bipolar affective disorders, depressive neurosis, and personality disorders.

The data linking positive and negative symptoms to later suicidal activity suggest a diagnosis-specific model for some risk factors. Positive symptoms may be suicide risk factors for some diagnostic groups and negative symptoms for other diagnostic groups, while poor functioning may be a general diagnoistic-free suicide risk factor.

Mann et al. [250] reviewed the literature and identified a number of strategies that are effective in the prevention of suicide such as education and awareness programs for the general public, primary care providers and other gatekeepers, screening for individuals at high risk, and providing treatment using pharmacotherapy and psychotherapy. In particular, the prevention of suicide in schizophrenia should include providing proper information for the family members of the patient in the hope of reducing their hostility toward the patient. In addition, continuity of care after suicide attempts, restricting access to lethal methods and media reporting guidelines are important strategies to prevent suicide. Since it is such a strong predictor of future suicide, preventing and reducing attempted suicide in schizophrenia may have a positive long-term impact.

Pompili et al. [251,252] reviewed the literature that dealt with the nursing of schizophrenic patients who are at risk of suicide These authors outlined key problems encountered in the nursing of these individuals, such as the unpredictability of suicide due to their fluctuating suicidal ideation, the staff's "countertransference" reactions to these patients, and the apparent improvement that precedes suicides. Nursing a schizophrenic patient who is at risk of suicide involves the establishment of a very unique relationship. Furthermore, the physicians' role in the prediction, prevention and management of suicide risk among schizophrenic patients should not be underestimated [253,254]. Family members are stigmatized for dealing with schizophrenia. This psychiatric disorder often results in impairment of daily activities, relapses and chronicity. Family members are viewed with suspicion as they cope with their sick relative, and they may be subjected to fewer social activities and reduced job opportunities. The family's difficulties and perceived stigmatization have been reported as possible contributing factors to the suicide of schizophrenic patients [255]. Finally, treatment professionals, as well as family members and other bereaved survivers of suicide, need encouragement to grieve and express their feelings about the suicide victim.

Pompili et al. [82] have recently stressed the need to implement prevention programs for suicide among schizophrenic patients. These authors focused on primary, secondary and tertiary prevention. Primary prevention represents the search for the prevention and the elimination of risk factors. These factors include social isolation, substance abuse, depression, hopelessness and disappointment for lost expectations for the future. Insight into the illness should be monitored very carefully as it has become apparent that the awareness of one's illness leads to discouragement and increased suicide risk. Appropriate pharmacotherapy and psychotherapy should prevent the emergence of risk factors for suicide and the reduction of those factors already detected in the patient. Patients should always be asked about their intention to commit suicide. There are no contraindications to the direct investigation of suicidality in schizophrenic patients. They are instead relieved by an explicit investigation as they have the opportunity to share their inner feelings [256].

Secondary prevention aims to check the phenomena in those subjects who have already developed risk factors for suicide. State-dependent risk factors are those that can potentially be modified (such as depression, substance abuse and hopelessness), while trait-dependent risk factors are unchangeable (such as gender, age and premorbid functioning). No doubt, a prompt recognition of individuals who are at risk is a key element in the prevention of suicide. Screening procedures taking into account suicidal indicators should be implemented. Patients who are depressed, substance abusers and hopeless should be monitored carefully. Those who have experienced multiple hospitalizations and previously threatened or attempted suicide should be treated with adequate procedures, such as programs of aftercare and psychosocial intervention.

Tertiary prevention is addressed to those individuals who have attempted suicide or have been suicidal in the past. Destigmatisation should be addressed to mental illness as well as suicide. Increasing the stigma associated with having suicidal feelings will increase the suicide rate. Interventions among families, mental health professionals and church activists aimed at decreasing the stigma associated with mental illness and suicide may contribute to the reduction of deaths by suicide. Pharmacological interventions are no doubt of paramount importance, but psychosocial interventions and psychoatherapy also play a central role.

This review has several limitations. It does not present mata-analytic results, and the authors adopted a narrative approach in order to summarise the information regarding suicide in schizophrenia. However, contributions were provided by scholars with an international reputation in this field. For this reason, this review differs from previous reviews and represents an original consensus conference approach from many authors who provided, on the basis of their expertise, a brief essay on specific aspects of the problem. References selected for this study may not include all of the works dedicated to the topic. Other key works may be available and may provide further understanding of the topic. Clearly, more joint efforts of this kind are needed to develop sound, shared guidelines for the prevention of suicide among individuals affected by schizophrenia.
